# INTEREST GROUP SESSION—HIV, AIDS AND OLDER ADULTS: HIV AND AGING: IMPROVING BIOPSYCHOSOCIAL HEALTH AND FUNCTIONAL WELLNESS

**DOI:** 10.1093/geroni/igz038.2020

**Published:** 2019-11-08

**Authors:** Monique J Brown, Molly M Perkins

**Affiliations:** 1 University of South Carolina, Columbia, South Carolina, United States; 2 Emory University School of Medicine, Atlanta, Georgia, United States

## Abstract

People living with HIV/AIDS (PLWH) are living longer due to the success of antiretroviral therapy. In 2016, approximately 50% of PLWH in the U.S. were age 50 and older and 17% of new infections were among this age group. One key factor that contributes to the HIV prevalence and incidence among older adults is the lack of sexual health discussion during healthcare visits. Among older (50+) PLWH, most are exhibiting signs of accelerated aging and declines in functional (physical, cognitive and psychosocial) wellness and little is known about the lasting impact of childhood sexual abuse (CSA). In this symposium, we present new research assessing the biopsychosocial challenges faced by older PLWH and older adults at risk for HIV. Dr. Robinson will present findings on the frequency, content and barriers and facilitators in provider communication with older patients on sexual health and HIV. Dr. Brennan-Ing will examine the association between CSA and psychosocial health among older PLWH. Dr. Schrack will assess the impact of energy utilization on physical activity and function among older HIV+ and HIV- men. Dr. Taylor will assess predictors of physical, cognitive and psychosocial wellness among older men with and without HIV. Dr. Sekhar will present findings from a trial on the impact of glycine and N-acetylcysteine supplementation on accelerated aging among older PLWH. Dr. Perkins, will discuss the public health implications of the presented research for HIV prevention and wellness interventions among older adults, and suggest future areas of inquiry to improve clinical outcomes for this population.

